# A hypoxia-related genes prognostic risk model, and mechanisms of hypoxia contributing to poor prognosis through immune microenvironment and drug resistance in acute myeloid leukemia

**DOI:** 10.3389/fphar.2024.1339465

**Published:** 2024-02-28

**Authors:** Xin Liu, Li Wang, Qian Kang, Cheng Feng, Jishi Wang

**Affiliations:** ^1^ Clinical Medical College, Guizhou Medical University, Guiyang, Guizhou, China; ^2^ School of Basic Medical, Guizhou Medical University, Guiyang, Guizhou, China; ^3^ Department of Hematology, Affiliated Hospital of Guizhou Medical University, Guizhou Province Institute of Hematology, Guizhou Province Laboratory of Hematopoietic Stem Cell Transplantation Centre, Guiyang, Guizhou, China; ^4^ National Clinical Research Center for Hematologic Diseases, The First Affiliated Hospital of Soochow University, Soochow, Jiangsu, China

**Keywords:** acute myeloid leukemia, hypoxia-related genes, risk prognostic model, immune microenvironment, drug resistance

## Abstract

**Objective:** Acute myeloid leukemia (AML) is a malignant hematologic cancer with poor prognosis. Emerging evidence suggests a close association between AML progression and hypoxia. The purpose of this study was to establish a new risk prognostic model for AML based on hypoxia-related genes, and to explore the mechanisms by which hypoxia-related genes affect the prognosis of AML based on tumor immune microenvironment (TIME) and drug resistance.

**Methods:** The AML patient samples obtained from Therapeutically Applicable Research to Generate Effective Treatments (TARGET) database were classified into C1 and C2 based on hypoxia-related genes, followed by analysis utilizing Gene Ontology (GO), Kyoto Encyclopaedia of Genes and Genomes (KEGG) and Gene Set Enrichment Analysis (GSEA). Through univariate and LASSO Cox regression analysis, the hypoxia-related hub genes 26S proteasome non-ATPase regulatory subunit 11 (*PSMD11*) and 26S proteasome non-ATPase regulatory subunit 14 (*PSMD14*) were identified to construct the model. AML patient samples were obtained from the TARGET and The Cancer Genome Atlas (TCGA) databases, serving as the training and the validation sets, and were stratified into high-risk and low-risk group according to the median risk score. The correlations between the model and TIME and anti-tumor drugs were analysed using CIBERSORT and Genomics of Drug Sensitivity in Cancer (GDSC) databases. The expressions of *PSMD11/PSMD14* in clinical samples and AML sensitive and drug-resistant cell lines were detected by Western blot and real-time PCR.

**Results:** The C1 group with high expression of hypoxia-related genes had lower overall survival (OS). Immune-related signaling pathways were different between C1/C2, and hypoxia was positively correlated with the activation of mammalian target of rapamycin (*mTOR*) signaling pathway. The model had good accuracy in both the training and the validation sets. The high-risk group exhibited lower OS and TIME activity, and was more sensitive to several anti-tumor drugs. *PSMD11/PSMD14* were highly expressed in relapsed patients and AML drug-resistant cell lines.

**Conclusion:** The established novel risk prognostic model and experiment results offer valuable insights for predicting AML prognosis and guiding drug selection. It also provides a fundamental framework for the mechanisms through which hypoxia impacts AML prognosis by modulating TIME and drug resistance.

## 1 Introduction

AML is a malignant clonal hematopoietic stem cell disease characterized by abnormal proliferation of primitive naive cells within the myeloid system (Döhner et al., 2015). It exhibits the highest incidence among adult leukemia cases (Estey and Döhner, 2006; Acute Myeloid Leukemia—Cancer Stat Facts, 2023). Despite achieving a complete response in many patients post-treatment, approximately half of them experience relapse. Notably, the 5-year survival rate decreases with age, with a mere 9% rate for individuals aged 65 and older (Miller et al., 2022). While traditional chemotherapy regimens and transplantation have been the standard treatment options, emerging therapeutic regimens such as novel targeted drugs, immunotherapy and cell therapy offer additional options for clinical treatment (Kayser and Levis, 2022). However, the recurrence rate of AML remains high and long-term survival remains low. Recently, with the wide spread adoption of second-generation sequencing and other technologies, the research on AML has gradually advanced (Newell and Cook, 2021), creating a new avenue to investigate the factors associated with the adverse prognosis of AML and its specific pathogenesis.

The bone marrow microenvironment (BMM) typically exists in a low oxygen state, maintaining physiological homeostasis through low blood partial pressure of oxygen (PO2). Hypoxia has conventionally been considered a niche characteristic that supports quiescence in hematopoietic stem cells (HSC) (Spencer et al., 2014). However, in AML, the malignant proliferation of leukemia stem cells (LSC) aggravates bone marrow hypoxia (Kosan and Godmann, 2016). In contrast to its role as a normal HSC environment, hypoxic BMM forms a “malignant niche” that fosters LSC survival and proliferation (Zhou et al., 2016). In this hypoxic BMM, LSC is induced with the ability to escape the cytotoxic effects of chemotherapy drugs, thereby acquiring drug resistance (Annaloro et al., 2011). Increasing evidence suggests that hypoxia provides an environment that promotes survival for AML cells, protecting them from apoptosis (Kremer et al., 2013; Wang et al., 2014). The study has indicated that hypoxia-mediated downregulation of Fms-like tyrosine kinase can result in cytarabine resistance *in vitro* AML cells (Sironi et al., 2015). The chemokine ligand 2 is capable of activating signaling pathways associated with AML cell survival, migration, and drug resistance under hypoxic conditions (Li et al., 2021b). Additionally, inhibiting hypoxia-induced histone deacetylase 9 expression contributes to the synergistic effect of venetoclax and MENIN inhibitor in KMT2A-rearranged AML (Ling et al., 2023). Therefore, there exists a close association between hypoxia and the malignant progression of AML.

The presence of hypoxia is a commonly observed hallmark in most solid tumor (Bai et al., 2022). Its presence not only hampers clinical efficacy, enhances tumor heterogeneity and drug resistance, but also disrupts TIME, thereby promoting tumor immune escape (Vito et al., 2020). Moreover, hypoxia-induced genes regulate diverse biological processes, enabling tumor cells to avoid apoptosis (Harris, 2002). Many known oncogenic signaling pathways overlap with hypoxia-induced signaling pathways (Hanahan and Weinberg, 2011), wherein their activation confers tumor cells with resistance to chemoradiotherapy and increased aggressiveness (Jing et al., 2019). Hypoxia also compromises the functionality of cytotoxic T cells and triggers the recruitment of regulatory cells, thus reducing the immunogenicity of tumors (Wu et al., 2022). In addition, it can stimulate tumor cells to secrete a substantial quantity of immunosuppressive molecules (Zheng et al., 2020), and regulate the number of immune checkpoint regulatory factors present on the cell surface (Noman et al., 2015). Overall, hypoxia directly inhibits the anti-tumor immune response, inducing immune escape and promoting tumor malignancy (Janker et al., 2019).

Drug resistance has been established as one of the main culprits of poor prognosis in AML (Estey, 2018), which can be attributed to multi-gene and multi-pathway interactions (Bolandi et al., 2021). Meanwhile, numerous studies have demonstrated that there are multiple internal and external immune escape mechanisms in AML (Vago and Gojo, 2020). For instance, immune escape changes in TIME promotes the malignant progression of AML (Christopher et al., 2018; Toffalori et al., 2019; Corradi et al., 2022). Nevertheless, whether hypoxia can influence AML prognosis through these two factors remain unclear and necessitates further exploration.

Collectively, hypoxia is considered an adverse factor affecting the prognosis of AML (Clarke and Fisher, 2019), as leukemic cells induce bone marrow hypoxia that results in the remodeling of the bone marrow niche (Li et al., 2021a). However, the effects of hypoxia on TIME and drug resistance in AML as well as the specific evaluation value of hypoxia-related genes in the clinical prognosis of AML remain unclear. Therefore, this study aims to establish a novel risk prognostic model for AML by utilizing hypoxia-related genes. Additionally, it explores the association between this model and TIME as well as anti-tumor drugs, and validates the correlation between hypoxia-related genes and drug resistance, consequently identifying the potential mechanism underlying hypoxia’s influence on the prognosis of AML. The establishment of such as model holds promise for future clinical treatments and drug selection.

## 2 Materials and methods

### 2.1 Clinical samples

The clinical samples were bone marrow samples collected from patients with AML and normal donors in the Department of Hematology, The Affiliated Hospital of Guizhou Medical University from 2021 to 2023. The patient’s condition was diagnosed using morphological, cytochemical, and immunotyping. Patients with AML were classified as “newly diagnosed” and “relapse” by diagnosis. Therefore, the clinical samples were divided into three groups: “normal donors” (n = 27), “newly diagnosed” (n = 33) and “relapse” (n = 29). Detailed data are shown in Supplementary Tables S1–S3. Written informed consent was obtained from the individuals for the publication of any potentially identifiable images or data included in this article. The clinical samples in this paper were approved by the Ethics Committee of the Affiliated Hospital of Guizhou Medical University for basic research, and the approval number is 2021 Ethics Approval No. 182.

### 2.2 Datasets

AML clinical samples were retrieved from TARGET (https://ocg.cancer.gov/programs/target) database as the training set, while data from the TCGA (https://www.cancer.gov/ccg/research/genome-sequencing/tcga) database were utilized as the validation set.

### 2.3 Data visualization

The sangerbox (http://sangerbox.com/) and the packages in R language mentioned below were employed for data visualization.

### 2.4 Consensus cluster analysis

**FIGURE 1:**
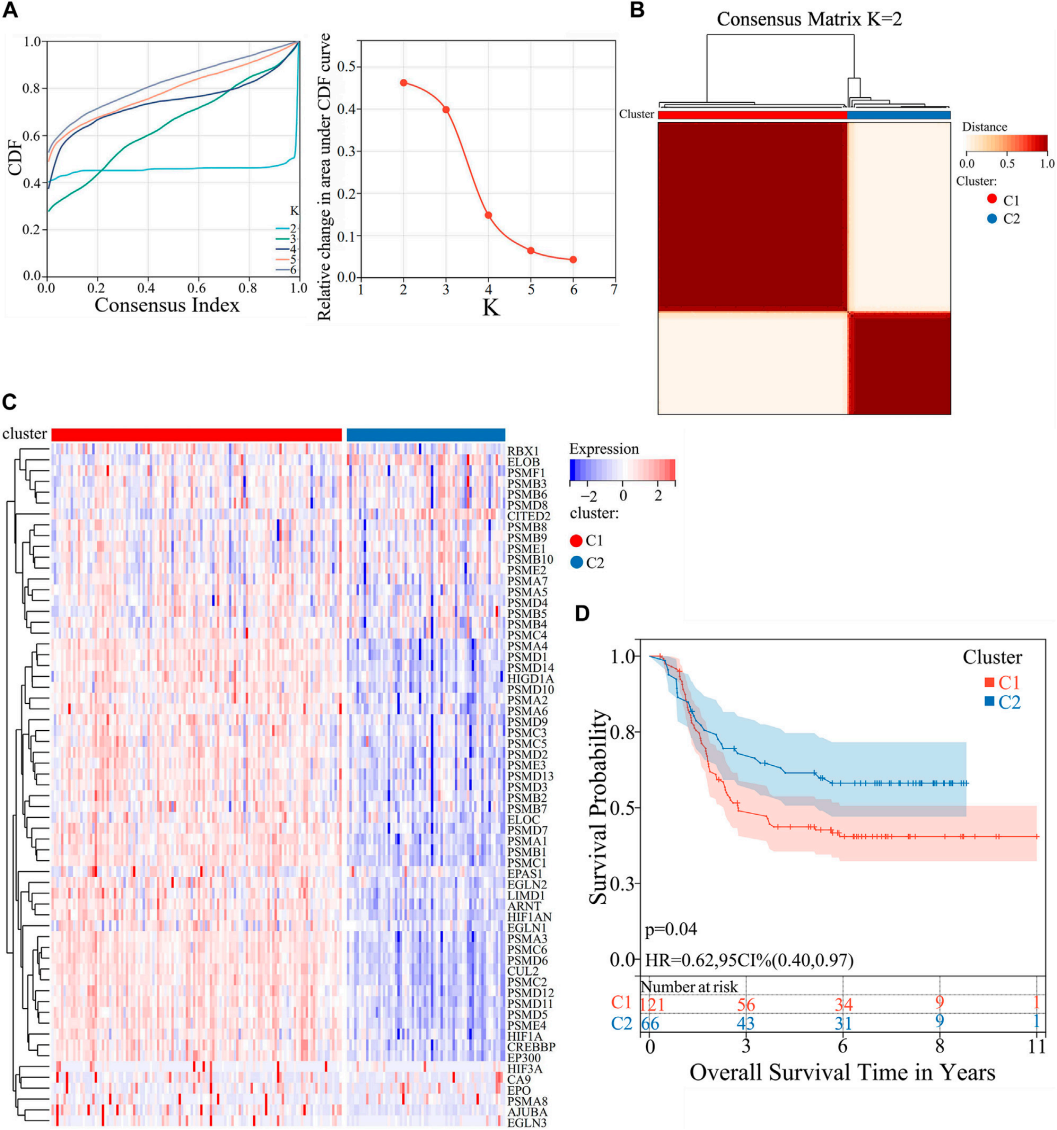
Consensus cluster analysis of AML patient samples based on the expression of hypoxia-related genes. **(A)** CDF curve and Delta area curve for K = 2–6. **(B)** Heatmap of consensus clustering matrix when K = 2. **(C)** Heatmap of the 63 hypoxia-related genes expression levels in C1 and C2. **(D)** KM curves of OS analysis for C1 and C2.

The “Consensus Cluster Plus” package in R language was applied for unsupervised consensus cluster analysis. Hypoxia signature genes were obtained from the Molecular Signatures Database (MSigDB) (https://www.gsea-msigdb.org/gsea/msigdb/) database. We selected ‘REACTOME_CELLULAR_RESPONSE_TO_HYPOXIA.v2022.1.Hs.gmt’ as our target gene set for the study. Hypoxia-related genes associated with AML clinical samples were screened using VLOOKUP function. Cluster numbers were between k = 2-6, and the relative area under the cumulative distribution function (CDF) curve were used to evaluate the clustering stability. Figures 1A–C was plotted using SangerBox.

### 2.5 Differentially expressed genes (DEGs) analysis

**FIGURE 2:**
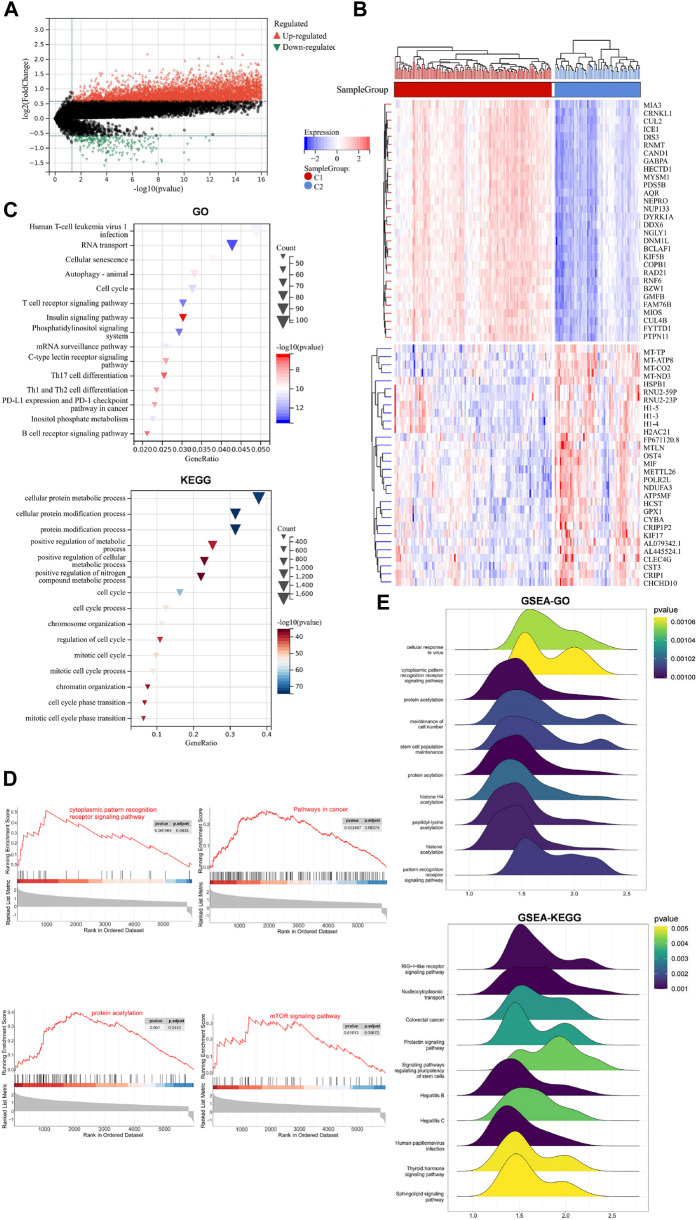
DEGs, related BP and signaling pathways in C1 and C2. **(A)** Volcano plot of DEGs in the two groups. **(B)** Heatmap of the DEGs expression levels in the two groups. **(C)** Bubble plots of the top 15 enriched BP and signaling pathways using GO and KEGG. **(D)** GSEA of the potential signaling pathways activated by hypoxia. **(E)** Ridge plot of the posterior distribution of GSEA-GO, and GSEA-KEGG.

The differential expression of mRNAs was evaluated using the “Limma” package in R language, with thresholds of *p* < 0.05 and log2|fold change| >0.585. The expression levels and distributions of DEGs between C1 and C2 were analyzed using the “Pheatmap” package in the R language. The sanger box was utilized to generate Figures 2A, B.

### 2.6 Survival analysis

**FIGURE 3:**
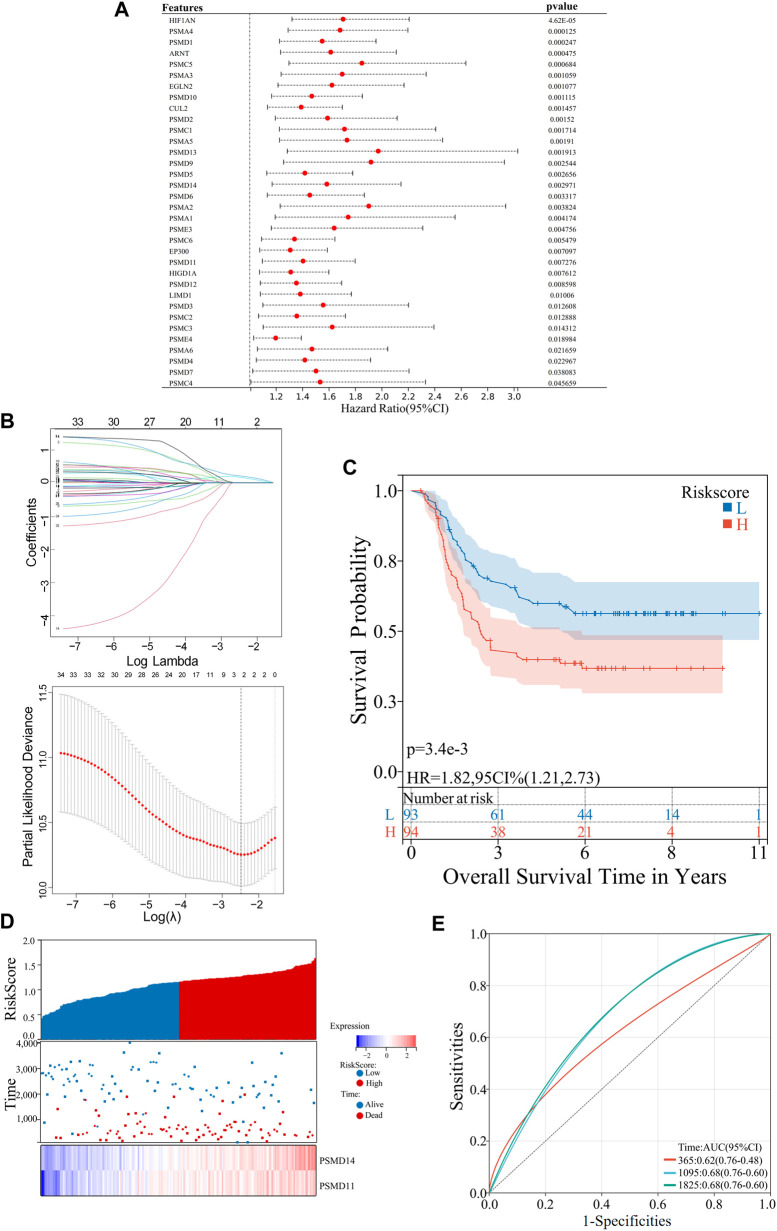
Construction of an AML risk prognostic model using hypoxia-Related Genes in the training set. **(A)** Forest plot of the univariate Cox regression analysis. **(B)** LASSO Cox regression exhibiting 2 hypoxia-related hub genes based on minimum λ = 0.09. **(C)** KM survival curves in the high- and low risk groups. **(D)** Distribution of the risk scores, scatter plot of the survival status, heatmap of *PSMD11* and *PSMD14* expression levels in the two groups. **(E)** ROC curves of the risk prognostic model predicting the prognosis of AML patients.

**FIGURE 4:**
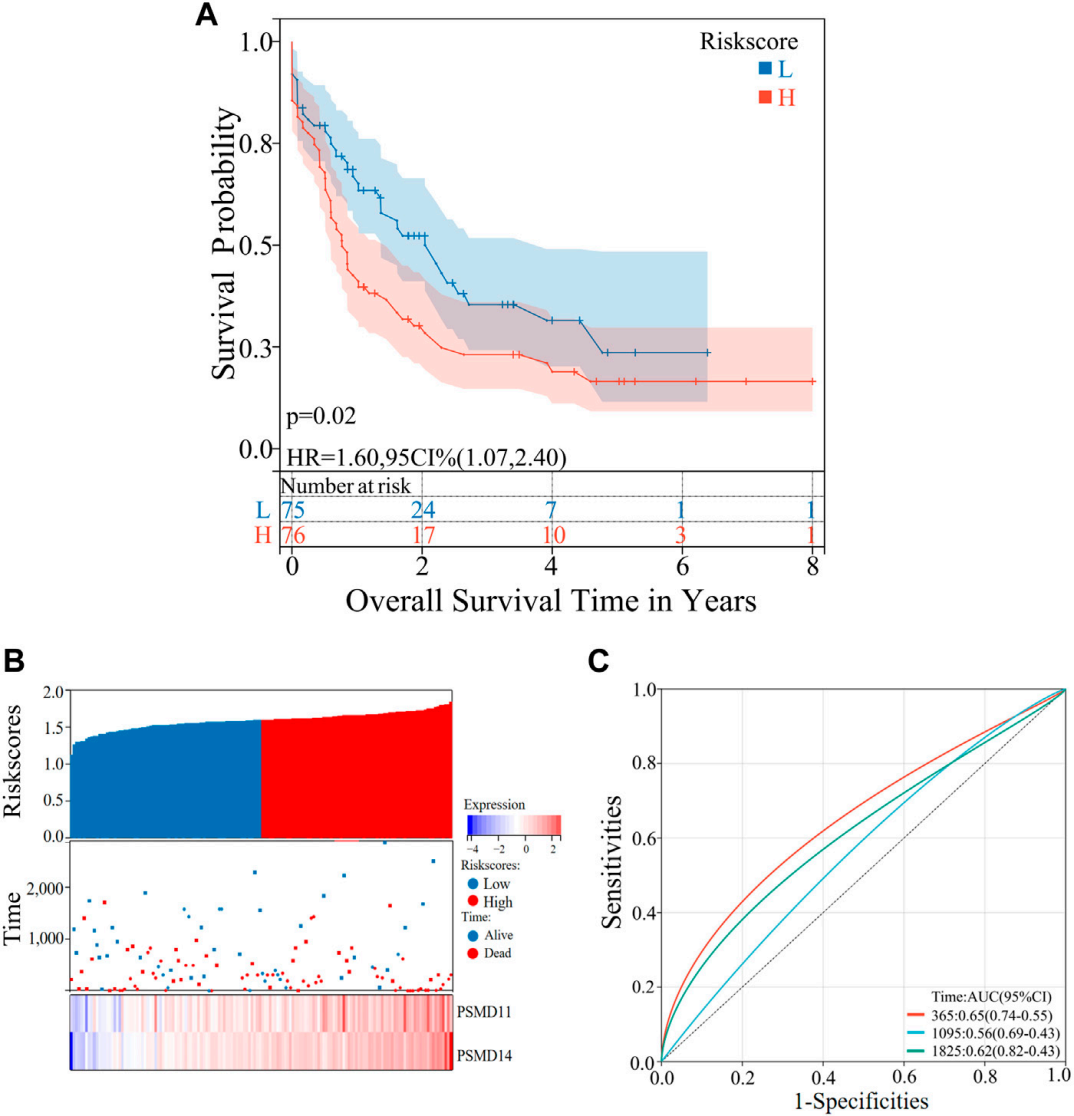
Validation of the risk prognostic models in the validation set. **(A)** KM survival curves in the high- and low-risk group. **(B)** Distribution of the risk scores, scatter plot of the survival status, heatmap of *PSMD11* and *PSMD14* expression levels in the two groups. **(C)** ROC curves of the risk prognostic model predicting the prognosis of AML patients.

The survival analysis was conducted using the “survival” package in the R language, and the OS of patients belonging to different clusters (C1 and C2) were analyzed and evaluated. Furthermore, a comparison was made between the 1-,3-and 5-year OS of high- and low-risk groups in both the training and validation sets. The Sangerbox was utilized for the visualization of Kaplan-Meier (KM) survival curves in Figures 1D, 3C, 4A.

### 2.7 Functional enrichment analysis

GO and KEGG were conducted using the “Cluster Profilter” package in R language, false discovery rate (FDR) < 0.05. Figure 2C was generated using sangerbox.

### 2.8 GSEA

The DEGs was subjected to pathway enrichment analysis using GSEA: (https:// www.broadinstitute.org/gsea/). Normalize enrichment score (NES): The normalized enrichment score after correction was normalized by the data of the gene set; NOM p-val: The *p*-value obtained by statistical analysis of ES value represents the reliability of the result; FDR q-val: The *p*-value after multiple hypothesis testing correction represents the probability of false positive results, The, the smaller the *p*-value, the more significant. |NES|>1, FDR <0.25, *p* < 0.05 was considered statistically significant. And “Cluster Profilter” in R language was used to draw Figures 2D, E.

### 2.9 Identification of hypoxia-related prognostic genes

Univariate Cox regression analysis was adopted to obtain 34 hypoxia-related genes that exhibited significant associations with OS in AML patients (hazard ratio, HR = 95%, *p* < 0.05). The “ggforest” package in R language was utilized to construct Figure 3A. LASSO Cox regression analysis was performed on 34 hypoxia-related genes to eliminate any false positive hypoxia-related genes that may be associated with prognosis. The “glmnet” package in R language was utilized to generate Figures 3B, 2 hypoxia-related genes were selected according to the minimum λ value for constructing the risk prognostic model.

### 2.10 Construction of the hypoxia-related risk prognostic model

The risk scoring formula is as follows:

$$\mathrm{Risk}\mathrm{score}=\sum_{i=1}^{n}\mathrm{coe}f_{i}*x_{i}$$

The term “Coefi” denotes the coefficient, while “Xi” represents the normalized count of each core gene. Receiver Operating characteristic (ROC) curve was generated using the R language package “time ROC”. The accuracy of the prognostic model in predicting the 1 -, 3 - and 5-year OS of AML patients was assessed by calculating the Area Under Curve (AUC) in both the training and validation datasets. Figures 3D, E was plotted using SangerBox.

### 2.11 Characterization of immune landscape

**FIGURE 5:**
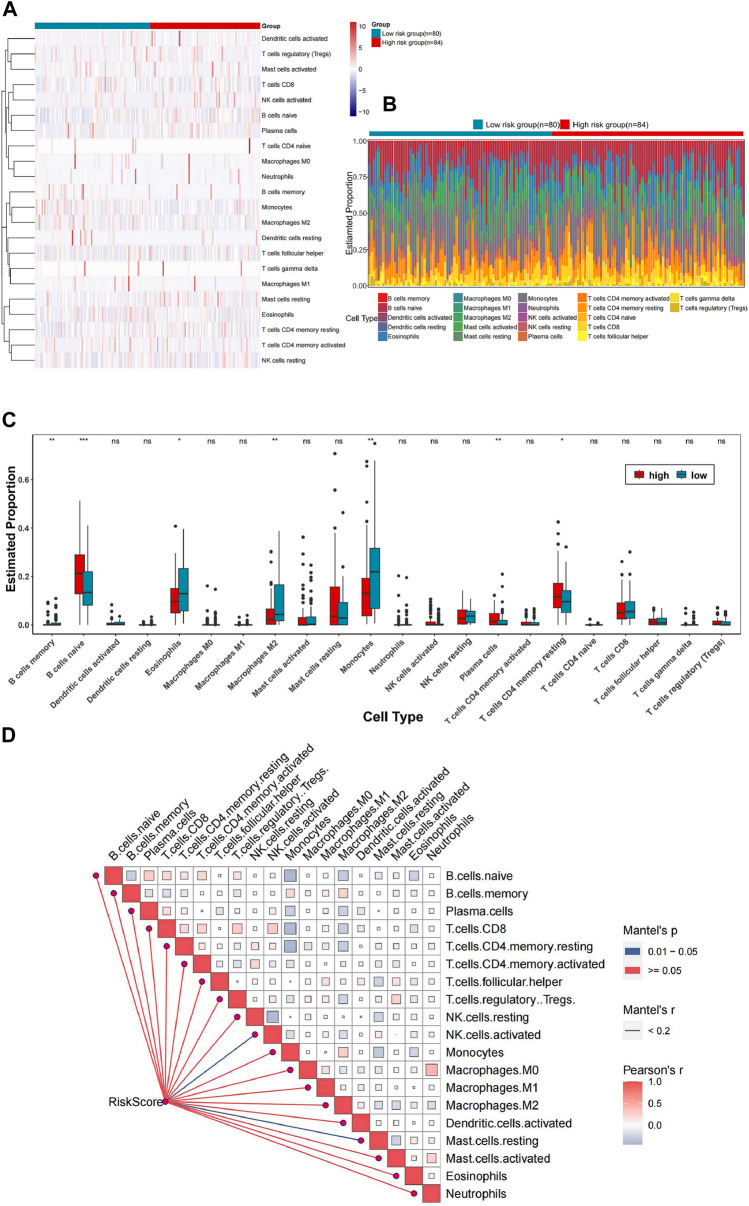
Correlation between the risk prognosis models and TIME. **(A)** Heatmap demonstrating the distribution of immune cells in the high and low-risk groups. **(B)** Stacked bar chart representing the overall calculation of immune cell infiltration in the two groups. **(C)** Box plots showing the infiltration of immune cells in both groups. **(D)** Correlation matrix of risk score matrix and immune cell matrix.

The CIBERSORT (https://cibersortx.stanford.edu/) in conjunction with the LM22 feature matrix was applied to analyze the differences in immune infiltration of 22 immune cells among different groups. The Pearson product-moment correlation coefficient was utilized to compute the correlation among immune cells, while the Mantel test was employed to statistically analyze the correlation between the risk score matrix and the immune cell matrix. r = 0-1 represents correlation, with higher values indicating stronger correlation, and *p* < 0.05 was considered statistically significant. The “CIBERSORT” package was used to conduct Figures 5A–C, while the “ggcor” package in R language was used to generate Figure 5D.

### 2.12 Drug sensitivity analysis

**FIGURE 6:**
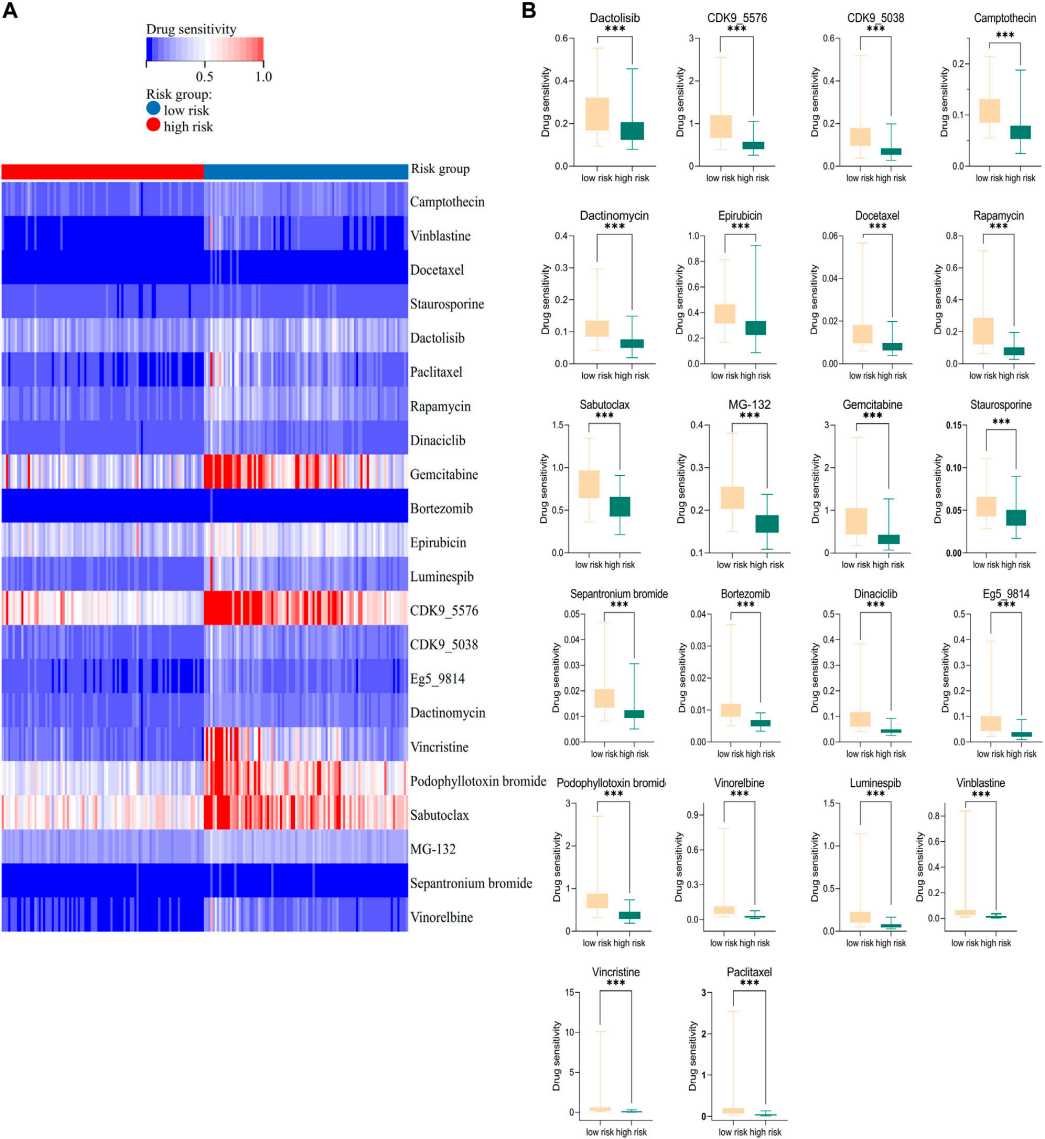
Association of the risk prognostic model with anti-tumor drugs. **(A)** Heatmap showing the distribution of the 22 anti-tumor drugs in high- and low-risk groups. **(B)** Box plot revealing the different drug sensitivities in the two groups.

The model was trained and drug sensitivity of the samples was predicted using ridge regression, based on the relationship between gene expression and drug IC_50_ in the training set. GDSC datebase preformed to analyze drug sensitivity of cancer cells. The sanger box was utilized to draw Figure 6A. The “oncoPredict” packages in R language were used to construct Figure 6B.

### 2.13 Extraction of bone marrow mononuclear cells from clinical samples

5 mL of bone marrow from AML patients or normal donors was collected by bone marrow puncture under routine sterile conditions and preserved with EDTA anticoagulation. Bone marrow was diluted 1:1 in equal volume with saline and was slowly added along the wall to a centrifuge tube pre-loaded with Ficoll (Solarbio Technologies, Beijing, China) separation solution, and Ficoll was 1:1 with diluted bone marrow. After centrifugation at 2,000 rpm for 15 min at room temperature, the intermediate white cell layer was aspirated and transferred to a new centrifuge tube. After centrifugation at 1,500 rpm for 5 min, the supernatant was discarded. The remaining precipitate, namely, bone marrow mononuclear cells, was retained after being washed three times with saline and subsequent discarding of the supernatant.

### 2.14 Cell culture

The authenticity of THP-1 and U937 human leukemia cell lines was confirmed by STR analysis, and they were cultured in a 5% CO_2_ incubator at 37°Cusing RPMI1640 medium containing 10% fetal bovine serum. Drug-resistant variants, namely, THP-1R and U937R, were generated by supplementing 1% penicillin (100 units/mL) and streptomycin (100 mg/mL) to the medium along with increasing concentrations of cytarabine (Ara-C). The drug concentration was gradually escalated, repeating this process three to five times at each concentration after the cells have proliferated to a normal shape. The drug induction was maintained for a duration of 6–8 months until the cells achieved a stable state at the final concentration.

### 2.15 Real-time PCR

The extraction of total RNAs from cells was performed using Trizol reagent (Invitrogen, Carlsbad, CA, United States). The mixture was vigorously shaken for 15 s after the addition of chloroform, followed by incubation at room temperature for 3 min. The samples were centrifuged at 12,000 rpm for 15 min at 4°C, and the supernatant was retained. An equal volume of isopropanol was added, followed by centrifugation at 12,000 rpm for 10 min at 4°C. Subsequently, the supernatant was discarded. The RNA precipitate was washed with 500 µL of 75% ethanol. Subsequently, the samples were subjected to centrifugation at a speed of 7,500 rpm for a duration of 3 min at a temperature of 4°C, followed by removal of the supernatant. The samples were air-dried for 5 min at room temperature to allow ethanol evaporation, followed by addition of DEPC water and measurement of concentration. cDNA was extracted using a reverse transcription kit (MedChemExpressMCE, United States of America). Real-time PCR was performed using SYBR Green PCR Master Mix (MedChemExpressMCE, United States) kit and PRISM 7500 real-time PCR Detection System (Thermo Fisher Scientific, United States). Relative expression of the target genes was calculated using *β-actin* as the reference through comparative cycle threshold (CT) values (2^−ΔΔCT^). The following human primers were used in this paper:

*β-actin* F 5′-CTA​CCT​CAT​GAA​GAT​CCT​CAC​CGA-3´;

*β-actin* R 5′-TTC​TCC​TTA​ATG​TCA​CGC​ACG​ATT-3´;

*PSMD11* F 5′-AGT​TCC​AGA​GAG​CCC​AGT​CT-3´;

*PSMD11* R 5′-TTG​CAC​TGC​CTC​TTC​ATC​GT-3´;

*PSMD14* F 5′- GTC​AGT​GTG​GAG​GCA​GTT​GAT​C-3′;

*PSMD14* R 5′-CCA​CAC​CAG​AAA​GCC​AAC​AAC​C-3′.

### 2.16 Western blot

The primary antibodies against PSMD11 and PSMD14 (Affinity Biosciences, United States of America) were diluted at 1:500 and 1:1,000, respectively, the β-actin primary antibody (Wuhan Sanying, China) was diluted at 1:3,000. Protein lysates were extracted from cells by adding 1 mM PMSF to the RIPA lysis buffer (Solarbio Science and Technology). The mixture was vigorously shaken and incubated on ice for 30 min. Subsequently, the supernatant was obtained by centrifugation at 12,000 rpm for 15 min at 4°C. The concentration of protein was determined using the BCA Protein Assay kit (Pierce, Hercules, CA, United States). The proteins were mixed with Loading buffer 1:4 and boiled at 100°C for 10 min 40 μg of proteins were then added to a 10% SDS-PAGE gel and electrophoresed into the separation gel at a constant voltage of 80 V followed by switching to a stable voltage of 120 V. At the end of electrophoresis, the separated proteins were transferred onto PVDF membranes and rotated at 250 mA for 1 h. After shaking with PBS containing 5% skim milk on a shaker for 2 h at room temperature, the membranes were washed. The primary antibodies were then incubated for more than 8 h at 4°C. After washing the membranes, secondary antibodies were incubated for at room temperature for 45 min. All protein bands were visualized using the Enhanced Chemistry kit (7Sea Biotech, Shanghai, China). β-actin was used as the internal reference.

### 2.17 Cell counting Kit-8 assay (CCK8)

CCK8 assay was used to detect the sensitivity of leukemia cell lines to Ara-C. The cells were inoculated into individual wells of a 96-well plate at a seeding density of 3 ×10^4^ cells/100 μL, with five replicates per experimental group. After subjecting the cells to various concentrations of Ara-c for a duration of 24 h, a volume of 10 μL CCK8 reagent was added into each well, the concentrations of Ara-c in U937 and U937R cell lines were 4, 16, 64, 192, 386, 578, 768 and 1,536 μM, meanwhile the concentrations of Ara-c inTHP-1 and THP-1R cell lines were 0.5, 4, 64, 192, 386, 578, 768 and1536 μM. The absorbance at 450 nm was quantified using a microplate spectrophotometer after co-culthring for 1–2 h. The IC_50_ value was determined by employing the GraphPad Prism 9.5 software.

### 2.18 Statistical analyses

The statistical software GraphPad Prism 9.5 was utilized for conducting both the analysis and visualization of data. The Shapiro-Wilk test and the Kolmogorov-Smirnov test were used to test the normal distribution of data. After the data passed the normal distribution test, the unpaired *t*-test was employed to compare and evaluate the differences between two groups. The experimental data were represented as mean ± standaed deviation (SD). The significance level, denoted by p calue, is interpreted as follows: *p < 0.05, **p < 0.01, ***p < 0.001 ****p < 0.0001; among these values, *p* < 0.05 is considered statistically significant.

## 3 Results

### 3.1 The AML patient samples were grouped using consensus cluster analysis based on hypoxia-related genes

To investigate the association between hypoxia and poor prognosis in AML, AML patient samples were grouped into differentsubtypes. Firstly, a search was conducted in the MSigDB database using the keyword “hypoxia”, resulting in 60 gene sets. After thorough consideration of factors such as the number of genes within each set, correlation with the research field, we ultimately identified a gene set containing 75 hypoxia-related genes as the target gene set. After screening using the VLOOKUP function, 63 of these genes were found to be linked to AML. Subsequently, unsupervised consensus cluster analysis was performed on 187 AML samples downloaded from the TARGET database, which were based on these 63 genes. The clustering stability is assessed by employing the CDF curve and Delta area curve for different cluster numbers (K = 2–6), the selection of K = 2 for clustering was presumed to be the optimal choice (Figure 1A). Accordingly, AML patient samples were classified into two subtypes, namely, C1 (n = 121) and C2 (n = 66) (Figure 1B). The heatmap revealed that the hypoxia-related genes in C1 displayed higher expression compared to those in C2 by analyzing the expression distribution of 63 hypoxia-related genes (Figure 1C). Therefore, C1 was defined as the high-expression hypoxia-related gene group, whereas C2 as the low-expression group. To explore the effects of hypoxia-related genes on the clinical prognosis of AML, survival analysis was performed in the two groups. The KM survival curve indicated a worse OS in the C1 group. (*p* < 0.05) (Figure 1D).

### 3.2 Identification of DEGs and signaling pathways between the different hypoxia subtypes

We screened out the DEGs between the two subtypes, GO, KEGG and GSEA were performed to analyse their biological functions and signalling pathways associated with hypoxia. This analysis allowed us to elucidate the mechanism by which hypoxia affects the clinical prognosis of AML. The “Limma” package in R language was utilized for filtering the DEGs between the two subtypes (|log2 FC|>0.585, *p* < 0.05). The volcano plot revealed 7,735 DEGs between C1 and C2, with 7,505 genes upregulated and 230 genes downregulated (Figure 2A). The heatmap, further revealed the expression level and distribution of DEGs between C1 and C2, (Figure 2B). GO and KEGG enrichment analyses of DEGs identified the top 15 relevant biological processes (BP) and signaling pathways (Figure 2C). Notably, the top three enriched of GO terms were the cellular protein metabolic process, cellular protein modification process and protein modification process. Parallelly, KEGG-enriched signaling pathways included human T-cell leukemia virus type 1 infection, the T cell receptor signaling pathway, *PD-L1* expression and the *PD-1* checkpoint pathway in cancer, TH17 cell differentiation and other immune-related signaling pathways.

To compensate for any omissions in the DEGs-based GO and KEGG analysis, especially owing to the threshold setting, and further elucidate the related signaling pathways that may be activated by hypoxia, GSEA was used to compare the gene sets (Figure 2D). GSEA revealed positive correlations between the high expression of hypoxia-related genes and the activation of several signaling pathways, including the cytoplasmic pattern recognition receptor signaling pathway (NES = 2.24), pathways in cancer (NES = 1.55), protein acetylation (NES = 2.21) and *mTOR* signaling pathways (NES = 1.68). The ridge plot presented the posterior distribution of GSEA-GO and GSEA-KEGG (*p* < 0.05) (Figure 2E), confirming significant enrichment in the cytoplasmic pattern recognition receptor signaling pathway and protein acetylation pathways, which are consistent with the previously enriched biological processes, such as protein modification processes.

### 3.3 Construction of an AML risk prognostic model using hypoxia-related genes

In further investigating the value of hypoxia-related genes in assessing the clinical prognosis of AML, a risk prognostic model of AML was constructed based on hypoxia-related genes. Initially, a univariate Cox regression analysis was performed to select 34 genes that were directly associated with the prognosis of AML from the 63 hypoxia-related genes (HR = 95%, *p* < 0.05) (Figure 3A). These 34 hypoxia-related genes were further analyzed using LASSO Cox regression to eliminate false positive factors related to prognosis. Using a minimum λ = 0.09, two hypoxia-related hub genes, *PSMD11 and PSMD14*, were finally selected to construct the risk prognosis model for patients with AML (Figure 3B). The risk scores for patients with AML were calculated by assessing the expression levels of *PSMD11* and *PSMD14* and applying risk coefficients according to the following formula: Risk Score = PSMD11*0.150603712635304+PSMD14*0.193834319424803.

The training set consisted of 187 AML patient samples downloaded from the TARGET database, which were further categorized into a high-risk group (n = 94) and a low-risk group (n = 93) based on the median risk score. Moreover, survival analysis was performed on the two groups, with the KM survival curve demonstrating a lower OS rate in the high-risk group (*p* < 0.05) (Figure 3C). Additionally, we analyzed the risk scores, survival status and *PSMD11* and *PSMD14* expression in the two groups. The high-risk group exhibited poorer clinical outcomes, whereas the expression levels of the two hub genes were significantly upregulated in this group (Figure 3D). Furthermore, the training set model was assessed using ROC-AUC to determine its accuracy. The AUC values for predicting the 1-, 3- and 5-year OS of patients with AML using this model were found to be 0.62, 0.68 and 0.68 respectively (Figure 3E). Thus, collectively, these results indicate that the hypoxia-related genes *PSMD11* and *PSMD14*-based AML risk prognostic model demonstrates good accuracy in predicting prognosis.

### 3.4 Verification of the predictive power of the hypoxia-related gene risk prognostic model

To confirm the reliability of the risk prognostic model, external dataset was employed for validation. A total of 151 AML clinical samples downloaded from the TCGA database was used as the validation set and were stratified into high-risk (n = 76) and low-risk (n = 75) groups based on the median risk scores. Similar to the training set, the KM survival demonstrated a lower OS in the high-risk group of the validation set (*p* < 0.05) (Figure 4A). Analyses were then conducted on the risk scores, survival status and core gene expression levels of AML patient samples in the validation set. The results obtained were consistent with those observed in the training set (Figure 4B). Finally, the AUC values of predicting the prognosis of 1-, 3- and 5-year AML patients were 0.65, 0.56 and 0.62, respectively (Figure 4C). This indicates that the model maintains its prediction ability in the validation set.

### 3.5 Association of the risk prognostic model with TIME of AML

The influence of hypoxia on the progression of AML through alterations in the TIME remains unclear. Therefore, the correlation between the risk prognostic model and the TIME of AML was investigated in this study. First, we utilized a heatmap to illustrate the distribution of immune cells between the high- and low-risk groups (Figure 5A). CIBERSORT combined with the LM22 characteristic matrix was adopted to analyze the disparity in immune cell infiltration between the two groups. Stacked bar chart represented the overall calculation of immune cell infiltration for each sample (Figure 5B). Additionally, the box plots demonstrated that the infiltration degree of B cells naive, mast cells resting and T cells CD4 memory resting was higher in the high-risk group, whereas that of eosinophils, macrophage M2 and monocytes was higher in the low-risk group (*p* < 0.05) (Figure 5C). These findings indicate that the activity of the immune microenvironment was lower in the high-risk group. Finally, the correlation between the risk score matrix and the immune cell matrix was statistically analyzed using Mantel test. The heatmap of the correlation matrix revealed a significant association between the risk score and NK cells as well as mast cells (0.01< Mantel’s *p* < 0.05, Person’r = 0–1) (Figure 5D).

### 3.6 Association of the risk prognostic model with anti-tumor drugs in AML

In order to explore the biological significance of the risk prognosis model, we performed drug predictions in the high- and low-risk groups. The “oncoPredict” package was utilized to identify 22 anti-tumor drugs associated with risk scores based on the GDSC database.The heatmap illustrated the distribution of drug susceptibility between the two groups (Figure 6A). The box plots of 22 drugs demonstrated that the high-risk group was more sensitive to the *mTOR* inhibitor rapamycin and the dual ATP competitive *PI3K* and *mTOR* inhibitor Dactolisib which were consistent with the results of GSEA. In other words, hypoxia in AML was found to activate the *mTOR* signaling pathway, with high-risk groups characterised by high expression of hypoxia-related genes, exhibiting higher sensitivity to *mTOR* inhibitors. Furthermore, the high-risk group also displayed lower resistance to protease receptor inhibitor bortezomib and protease inhibitor MG132, suggesting that these drugs are effective in patients with high expression of *PSMD11* and *PSMD14*, components of the 26S proteasome complex. (*p* < 0.05) (Figure 6B). Consequently, these anti-tumor drugs may yield better therapeutic outcomes in the high-risk group. Collectively, the risk prognosis model holds biological significance.

### 3.7 *PSMD11* and *PSMD14* were closely associated with poor prognosis of AML

**FIGURE 7:**
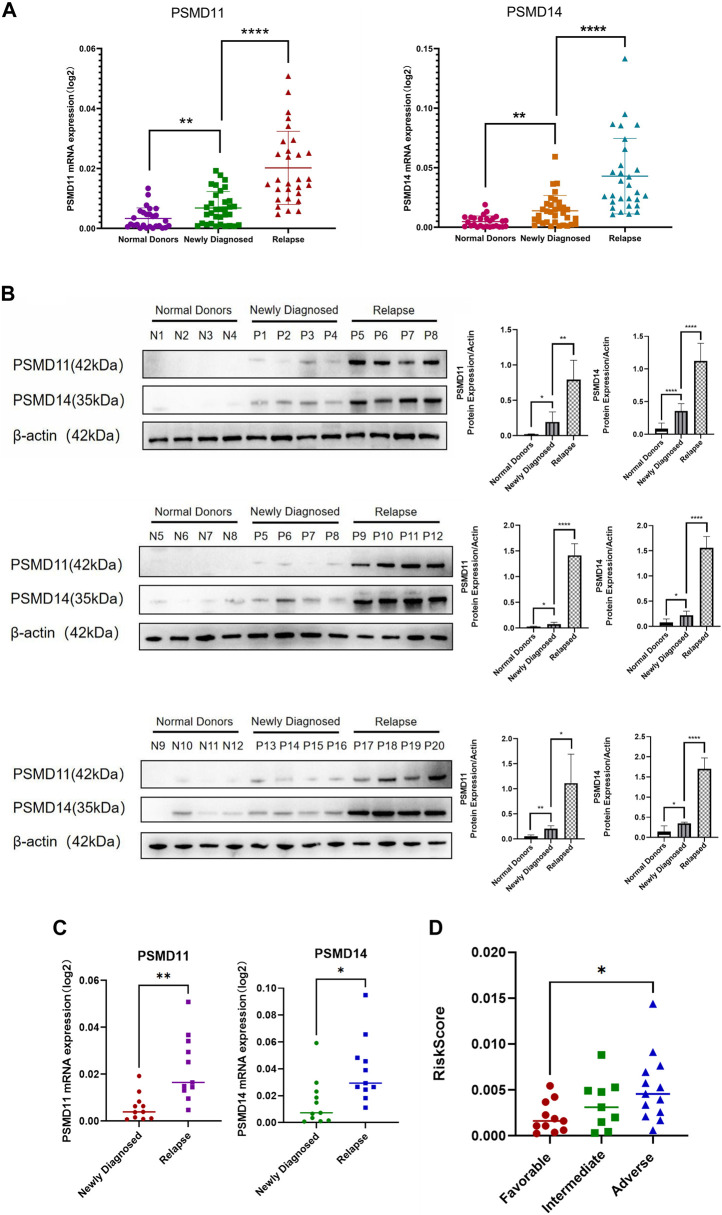
Association of *PSMD11* and *PSMD14* with the poor AML prognosis. **(A)** The mRNA expression of *PSMD11* and *PSMD14* in the “normal donors group” (n = 27), “newly diagnosed group” (n = 33) and “relapse group” (n = 29). **(B)** The protein expression of PSMD11 and PSMD14 in the “normal donors group” (n = 12), “newly diagnosed group” (n = 12) and “relapse group” (n = 12). Grey values of *PSMD11*, *PSMD14*. **(C)** mRNA expression of *PSMD11* and *PSMD14* at newly diagnosed and relapse in the same patient (n = 11). **(D)** Risk scores for the “favorable group” (n = 11), “intermediate group” (n = 9), “adverse group” (n = 13).

To further verify the influence of hub genes *PSMD11* and *PSMD14* in the risk prognostic model on patient prognosis, their expressions in bone marrow blood samples of AML patients and normal donors were examined. Real-time PCR and Western blot results revealed that the mRNA and protein expression levels of *PSMD11* and *PSMD14* were highest in the relapse group and lowest in the normal donors group, with significantly higher levels observed in the relapse group compared to the newly diagnosed group (*p* < 0.05) (Figures 7A, B). Notably, within a subset of 11 patients providing both newly diagnosed and relapse samples, the mRNA expression of *PSMD11* and *PSMD14* were consistently higher in the relapse group than that in the newly diagnosed group of the same patient (*p* < 0.05) (Figure 7C). Then, we divided the newly diagnosed clinical samples into 3 risk categories as favorable, intermediate, adverse groups based on the updated ELN AML Guidelines for 2022 (Döhner et al., 2022), and calculated risk scores for these clinical samples. The analysis revealed that the risk scores of clinical samples in the adverse group was significantly higher than those of the favorable group (*p* < 0.05) (Figure 7D). Collectively, these results further confirm the reliability of the risk prognosis model, and highlighted the close association of *PSMD11* and *PSMD14* with the poor AML prognosis.

### 3.8 *PSMD11* and *PSMD14* were highly expressed in AML drug-resistant cell lines

**FIGURE 8:**
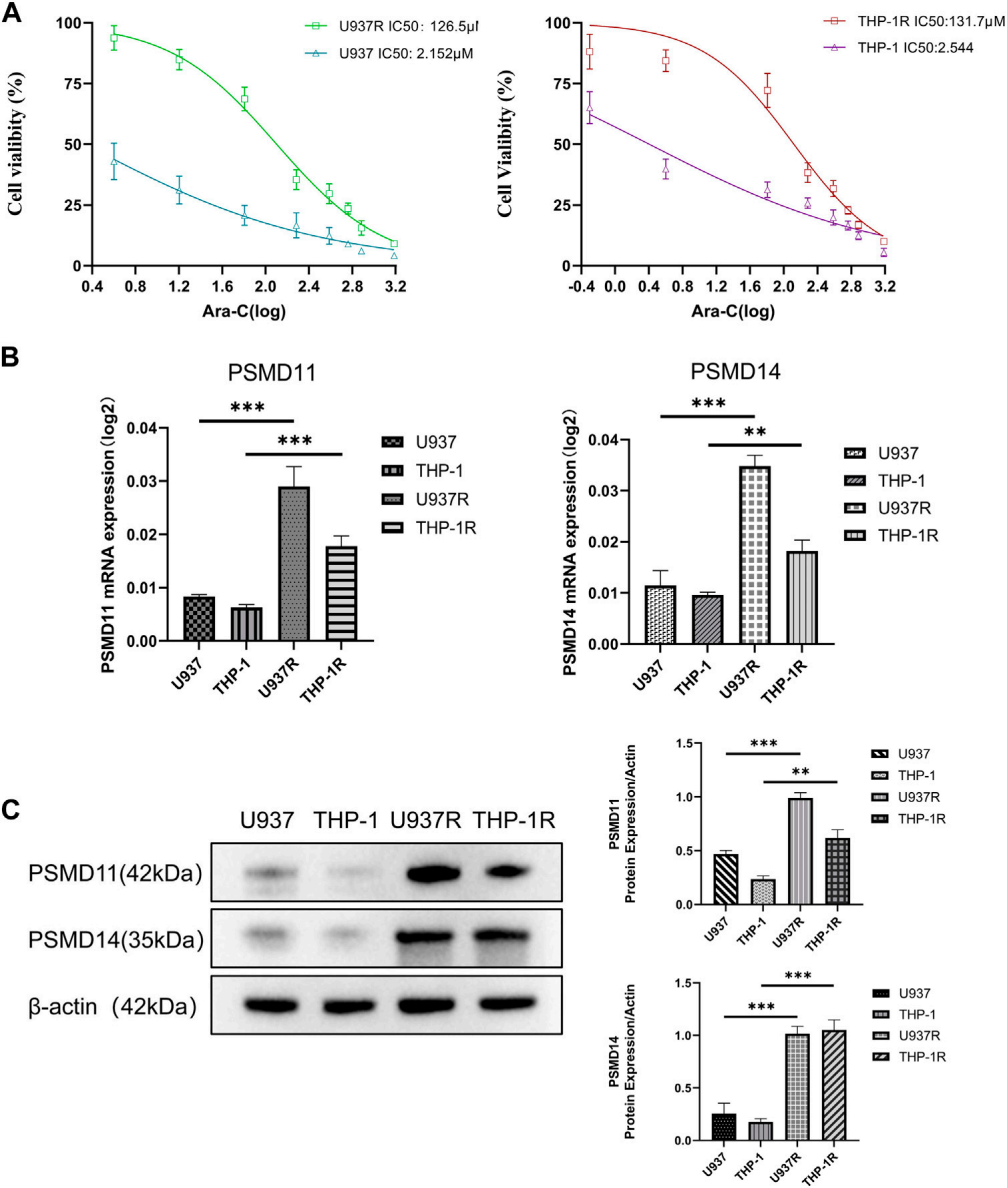
High expressions of *PSMD11* and *PSMD14* in AML drug-resistant cell lines. **(A)** The cell viability of U937, U937R, THP-1 and THP-1R cell lines. **(B)** The mRNA expression of *PSMD11* and *PSMD14* in AML cell lines. **(C)** The protein expression of PSMD11 and PSMD14 in AML cell lines. Grey values of *PSMD11*, *PSMD14*. The cell experiments were independently repeated three times for each trial.

Finally, to confirm the association between the risk prognostic model and the resistance towards conventional chemotherapeutic drug, we assessed the viability of AML-sensitive cell lines U937 and THP-1, as well as drug-resistant cell lines U937R and THP-1R under varying concentrations of Ara-C was examined using CCK8. The IC_50_ values of U937 and THP-1 were 2.152 and 2.544 μM, whereas those of U937R and THP-1R were 126.5 and 131.7 μM, respectively, indicating that U937R, THP-1R cells were 58.8 and 51.8 times more resistant than U937and THP-1 cells (Figure 8A). Real-time PCR and Western blot results showed that the mRNA and protein expression levels of *PSMD11* and *PSMD14* were higher in AML drug-resistant cell lines (*p* < 0.05) (Figures 8B, C). These findings provide evidence that elevated levels of *PSMD11* and *PSMD14* are closely related to AML drug resistance, indicating a potential role of hypoxia in contributing to poor AML prognosis through drug resistance.

## 4 Discussion

In this study, we focused on the role of hypoxia and its impact on drug resistance and TIME in the prognosis of AML. Therefore, we constructed a new risk prognostic model of AML using hypoxia-related genes, namely, *PSMD11 and PSMD14*. This model demonstrated good prognostic accuracy and provided a novel reference for the clinical prognosis prediction of AML. We have also observed that hypoxia can induce drug resistance and TIME suppression in AML, which providing novel insights into the mechanism underlying a poor prognosis in this disease. Moreover, the enrichment analysis of GO and KEGG revealed that the BP and signaling pathways were primarily associated with protein modification and immune-related signaling, indicating a potential role for hypoxia in promoting the occurrence and progression of AML through modulation of protein modification and alteration of TIME. Additionally, our results revealed that *mTOR* signaling pathway may be activated by hypoxia-related genes in AML. It is known that *mTOR* as a pivotal regulator of cellular metabolism, exerts its control over cell growth and proliferation via diverse signaling pathways (Kim and Guan, 2015). Studies have suggested that the *mTOR* signaling pathway is activated in many tumors, thereby promoting tumor growth and proliferation and inducing drug resistance (Mossmann et al., 2018; Hua et al., 2019). *PI3K*-*Akt*-*mTOR* activation is currently known a poor prognostic factor in AML (Nepstad et al., 2020). Therefore, further investigation is warranted to elucidate the mechanism by which hypoxia activates *mTOR* signaling and consequently contributes to unfavorable prognosis in AML.

The 26S proteasome comprises a single catalytic 20S complex (core particle) and one or two 19S regulatory complexes (regulatory particle), which catalyzes the degradation of most proteins and plays a crucial role in numerous cellular processes (Collins and Goldberg, 2017). The 19S regulatory complex is responsible for the deubiquitination, unfolding and reorientation of proteins to catalytic sites within the 20S complex (Budenholzer et al., 2017). After recognizing ubiquitination labeled proteins, 26S proteasome can degrade unnecessary or damaged thereby playing a proteins by a crucial role in maintaining cell homeostasis (Collins and Goldberg, 2017). Hypoxia-related genes *PSMD14* and *PSMD11* are components of the 19S subunit (Hoffman and Rechsteiner, 1997; Sun et al., 2021). *PSMD11*, also known as *RPN-6*, plays a pivotal role in the regulation of both the assembly and activity of 26S proteasome (Vilchez et al., 2012) and its high expression or phosphorylation promotes 26S proteasome assembly and enhance proteasome activity (Lokireddy et al., 2015). Studies have revealed that *PSMD11* serves as a potential biomarker for predicting the progression of pancreatic cancer (Sahni et al., 2020). Moreover, the rapid synthesis of the PSMD11 protein is related to the activation of the *MEK1/ERK1/2* signaling pathway (Wang et al., 2018). *PSMD14* is also known as *POH1/Rpn11*, and its high expression is associated with tumor progression, high tumor grade, reduced susceptibility to cytotoxic drugs, and poor prognosis (Spataro and Buetti-Dinh, 2022). Numerous studies have associated *PSMD14* with the occurrence and development of various tumors (Luo et al., 2017; Song et al., 2017; Yu et al., 2019; Zhang et al., 2020a; Lv et al., 2020; Jing et al., 2021). Notably, bortezomib, a proteasome inhibitor, has been used in the clinical treatment of multiple myeloma (Goldberg, 2012). The newly developed *PSMD14* inhibitors, capzimin and thiolutin, also demonstrated robust anti-cancer activity in solid tumors and leukemia cell lines (Spataro and Buetti-Dinh, 2022). And *PSMD14* inhibition can induce multiple myeloma cell apoptosis and overcome bortezomib resistance (Song et al., 2017). Thus, *PSMD14* considered a drug target for cancer treatment, especially in the context of inhibiting cancer progression (Bustamante et al., 2023). In general, *PSMD11* and *PSMD14* are closely related to tumor development and poor prognosis, however few studies in the context of AML are lacking. Therefore, it is necessary to continue exploring the mechanism of *PSMD11* and *PSMD14* in AML. In this study, the *in vitro* cell experiments revealed a potential mechanism by which hypoxia may lead to AML drug resistance through overexpression of *PSMD11/PSMD14*. Moreover, the analysis of anti-tumor drugs suggested that the AML patients exhibiting high levels of *PSMD11/14* expression may benefit from utilizing *mTOR* inhibitors or proteasome inhibitors. Thus, *PSMD11/PSMD14* can be considered potential biological markers of AML drug resistance, and the drug sensitivity analysis results have the potential to guide drug selection for patients with AML.

The immune microenvironment landscape analysis under immune cell infiltration has been widely used in tumor research, analysis of the effect of the immune microenvironment on tumors aids in the advancement of immunotherapy (Zhang et al., 2020b; Klemm et al., 2020). Our study revealed that the immune microenvironment exhibited diminished activity in the high-risk group, therefore, we speculated hypoxia as a contributing factor to the suppression of TIME in acute myeloid leukemia AML. Moreover, strong correlations between the risk score and NK cells as well as mast cells were observed, suggesting that hypoxia may exert immunosuppressive effects in AML by modulating these immune cell. Among them, mast cells secrete a variety of cytokines, participate in immune regulation by activating antigen-presenting cells (APCs), express major histocompatibility complex (MHC) molecules, *B7* molecules, and also function as APCs (Komi and Redegeld, 2020). However, NK cell-mediated tumor recognition is independent of MHC, but relies on the interaction of inhibiting and activating receptors on NK cells and several ligands on the surface of tumor cells (Poznanski and Ashkar, 2019). Our research team has reported that Heme Oxygenase-1 (*H O -1*) induces NK cell dysfunction and promotes the occurrence and development of AML (Zhang et al., 2022; Feng et al., 2023). mRNA and protein expressions of *H O -1* are established to be upregulated in response to oxidative stress and cell damage (Chiang et al., 2021). Meanwhile, hypoxia and oxidative stress also interact with each other. During the process of cell metabolism, hypoxia induces a series of reactions to produce oxidative substances such as free radicals and thereby promote cell damage (You et al., 2021). Therefore, we hypothesize that hypoxia may regulate the expression of *H O -1* by influencing oxidative stress, thus inhibiting the activity of NK cells and ultimately leading to the poor prognosis of AML.

Finally, due to the utilization of publicly available databases for model construction in this study, it lacks certain accuracy. Nevertheless, the clinical sample experiments showed that the high expression of *PSMD11/PSMD14* was associated with poor prognosis in AML which further confirmed the reliability of the risk prognosis model. Additionally, further experimental verification of the specific mechanism of hypoxia leading to immunosuppression elucidated herein is required, which is the limitation of this study and the future research direction of our research team.

## 5 Conclusion

In this study, a robust and novel risk prognostic model of AML was constructed using hypoxia-related genes, namely, *PSMD11* and *PSMD14*, which showed good prognostic accuracy. Moreover, hypoxia has been observed to induce TIME inhibition in AML, and to affect the malignant progression of AML through the activation of the *mTOR* signaling pathway and overexpression of *PSMD11* and *PSMD14*. Anti-tumor drug analysis revealed that patients in the high-risk group were more sensitive to *mTOR* inhibitors and proteasome inhibitors. Furthermore, clinical sample analysis and *in vitro* cell experiments confirmed the reliability of the model and the correlation between hypoxia and drug resistance. Thus, the established risk prognostic model offers valuable insights for predicting AML prognosis and guiding drug selection. It also sheds light on the mechanisms by which hypoxia contributes to poor AML prognosis through TIME disruption and drug resistance.

## Data Availability

The original contributions presented in the study are included in the article/Supplementary Material, further inquiries can be directed to the corresponding author.
